# Socio-ecological influences of adolescence marijuana use initiation: Qualitative evidence from two illicit marijuana-growing communities in South Africa

**DOI:** 10.4102/sajpsychiatry.v26i0.1477

**Published:** 2020-08-26

**Authors:** Emmanuel Manu, Mbuyiselo Douglas, Martin A. Ayanore

**Affiliations:** 1 Department of Population and Behavioural Sciences, School of Public Health, University of Health and Allied Sciences, Ho, Ghana; 2 School of Nursing and Public Health, University of KwaZulu-Natal, Durban, South Africa; 3 Department of Health Policy Planning and Management, School of Public Health, University of Health and Allied Sciences, Ho, Ghana

**Keywords:** socio-ecological model, substance abuse, marijuana use, adolescence, Ingquza Hill local municipality, South Africa

## Abstract

**Background:**

Adolescence has been identified as a critical risk period for substance use initiation, such as marijuana. Although several factors have been cited for adolescent marijuana use, those that influence initiation, especially in an African setting where illicit marijuana activities are rife, have not been contextually explored.

**Aim:**

We ascertained the factors that influence adolescent marijuana use initiation in two marijuana-growing communities in the Eastern Cape province of South Africa, based on the constructs of the socio-ecological model.

**Setting:**

The study was conducted in two selected illicit marijuana growing communities in the Ingquza Hill Local Municipality of the Eastern Cape province of South Africa.

**Methods:**

Focus group discussions (FGDs) were conducted among 37 participants, grouped into four focus groups. Purposive and snowball sampling techniques were used to select the communities and participants, respectively. An FGD guide was used to collect the data. The data were analysed using thematic content analysis approach and presented under various themes.

**Results:**

Twelve influences of adolescent marijuana use initiation, grouped under three main levels of socio-ecological influence, personal characteristics (curiosity, shyness and fulfilment of personal need), micro-level influences (peer pressure, negative school climate, presence of marijuana in households and parental or sibling marijuana use) and macro-level influences (child labour, poverty, presence of marijuana in communities, presence of negative adult role models and breakdown in communal restrictions against marijuana use), were found.

**Conclusion:**

Health promotion programmes, targeting socio-ecological motives of adolescent marijuana use initiation in the two communities, should be intensified to break the cycle of adolescent marijuana use. Also, alternative livelihood schemes should be implemented in the affected communities to break the cycle of illegal marijuana cultivation that promotes adolescent marijuana use.

## Introduction

Adolescent marijuana use is a widespread global public health challenge as marijuana is the most commonly used illicit drug and the drug of choice for initiation into the use of other illicit drugs.^1,2^ Adolescence has been identified as a critical risk period for substance use initiation.^3^ While marijuana use initiation often starts in the late adolescence to early adulthood, those who do so in their early adolescence face the risk of acute harm and increased susceptibility to developing drug use disorders as well as mental health disorders, including personality disorders, anxiety and depression.^4^ Despite its debilitating mental health effects,^4,5^ adolescent marijuana use continues to be a worldwide problem. In the United States, for instance, the prevalence of marijuana use among adolescents is so dire that as far back as in 2010, 13-year-old children were found to be using the substance.^6^ Similar patterns have been found in the United Kingdom where 3.4% of adolescents were reported to be regular users of the marijuana, with factors such as peer influence and maternal substance use found to be associated with adolescence marijuana use initiation.^7^

**FIGURE 1:**
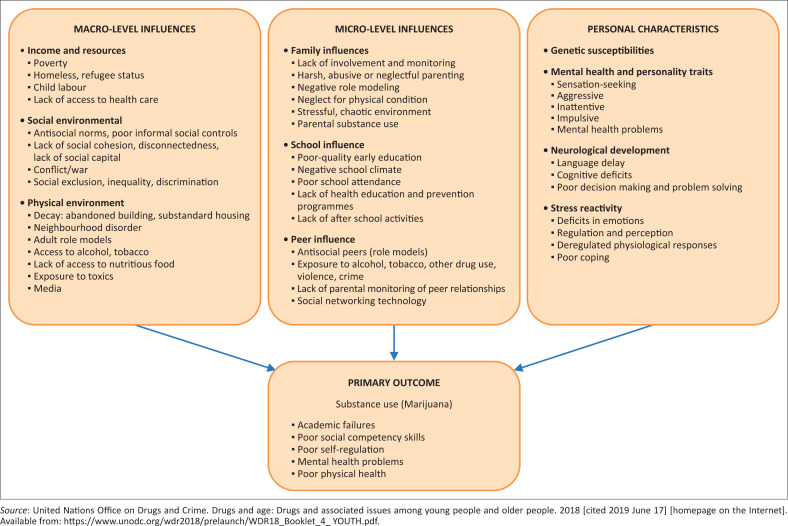
Socio-ecological model of factors influencing adolescence substance use.^1^

The problem of adolescent marijuana use is not different in South Africa. According to the Cancer Association of South Africa,^8^ marijuana use is very rife among South African adolescents. To increase the vulnerability of adolescent marijuana use, communities in the Ingquza Hill Local Municipality, which are part of the former Pondoland region, are known for extensive illegal marijuana cultivation and trading for generations.^9,10^ This therefore exposes adolescents to marijuana use and its associated mental and psychiatric harm.^5^ Thus, factors that influence adolescent marijuana use could be found in their immediate environment. Theoretically, the United Nations Office on Drugs and Crime Prevention (UNODC) has grouped risk factors of adolescent illicit substance use into three broad categories: personal level influence (genetic susceptibilities, mental health and personality traits, neurological development and stress reactivity), micro-level influences (family influences, school influences and peer influences) and macro-level influences (income and resources, social environment and physical environment).^1^ These factors are comprehensively presented in Figure 1.

In South Africa, qualitatively explored influences of adolescent marijuana use initiation are scarce^11^ as most studies generally focus on quantitative exploration of predictors and trends of adolescence substance use.^12,13,14,15,16^ Recently, a study conducted by Francis et al.^17^ only looked at the link between religiosity, alcohol and other drug use among young adolescents in the Western Cape province of South Africa. Thus, despite the abundance of the literature on adolescent substance use in the country, including marijuana, contextual influences of adolescent marijuana use, focusing on the various socio-ecological initiation pathways, particularly among adolescents in vulnerable populations,^18^ such as in the Ingquza Hill Local Municipality of the Eastern Cape province, remain empirically unascertained. We therefore looked into initiators of adolescent marijuana use in South Africa, based on the tenets of the UNODC’s socio-ecological model, in two selected communities of the Ingquza Hill Local Municipality of South Africa, where illicit marijuana cultivation and trading is pronounced. This was to help understand the implication of these factors on public health promotion programmes aimed at addressing illicit drug use, especially marijuana use, among adolescents in communities with high level of availability of illicit drugs in South Africa and in similar settings as a whole.

## Methods

### Study setting

The study was conducted in two illegal marijuana-growing communities, Community 1 and Community 2, in the Ingquza Hill Local Municipality of the Eastern Cape province of South Africa.^9,10^ The Municipality has an estimated total population of 278 481 people with a population density of 234 people per square kilometre.^11,19^ The communities were purposively chosen for the study because they were known to be involved in illegal marijuana cultivation and trading^9,10^ as such adolescents were ecologically exposed to marijuana. The communities were therefore deemed to be information-rich in answering the research question, hence their purposive selection for the study.

### Research design

The focus group discussion (FDGs) approach was adopted in this study. Focus group discussions have been described *as a thinking society in miniature* because they observe the co-construction of meaning and the development of knowledge at the broader group level through group interaction.^20^ The interactions within the group allow researchers a controlled first-hand experience of how the society processes and makes sense of social phenomenon.^20^ Our focus groups consisted of people who knew each other and shared similar experiences in terms of exposure to marijuana and its usage. The choice of focus groups as a method allowed the researchers to observe areas of strong emphasis, especially in aspects that discussants were most passionate about.^21^ Moreover, FGDs led to group interactions that produced data and insights that would have been less accessible without group interactions.^22^ Adolescents aged 14–19 years, who resided in either of the two communities since their childhood and assented to participate in the study, upon parental consent, were recruited for the study.

### Population and sampling

A total of 37 participants aged 14–19 years from two communities, Community 1 and Community 2, grouped into two focus groups per community, were involved in the study. Two focus groups per community (thus four in total) were formed based on the number of participants who agreed to participate in the study from each community, taking into account societal stigmatisation associated with the topic of discussion, marijuana smoking.^23,24^ Thus, the number of adolescent marijuana smokers who agreed to participate in the study from each community only allowed for the formation of two focus groups per community.^22^ Assignment of participants to a group was performed based on familiarity and trustworthiness of other participants. Thus, participants who smoked marijuana together with their known and trusted peers were grouped together. Because of the stigmatisation of marijuana smokers, participants smoked marijuana in secrecy in a form of a cult. Hence, grouping same the cult members in the same discussion group brought about some sense of security and secrecy that opened them up to freely share their views. Although grouping was based on familiarity and trustworthiness, the four groups were comparable in the sense that all participants were adolescents, were active marijuana smokers and also shared similar socio-cultural characteristics. Thus, their behaviour was shaped and influenced by similar environmental constructs which made their views and experiences comparable.^25^ A two-staged non-probability sampling procedure was followed to recruit participants for this study.

First, purposive sampling was used to select the two communities from a list of communities under the Ingquza Hill Local Municipality, as the variable of interest was communities that cultivated marijuana illegally. Thus, two communities where marijuana was known to be illegally grown were purposively chosen as the study sites. In order not to expose or portray participants to their legal guardians as marijuana smokers, verbal permission was sought from community members (including guardians) for participants who were below 18 years, through community tribal courts, where the nature and purpose of the study was explained to the general public.

After permission was granted to conduct the study in the communities, a key informant identified in each community after staying in each community for a period of 2 weeks aided in the recruitment of participants known to be marijuana smokers in the communities using the snowball sampling technique. Thus, once an adolescent marijuana smoker was successfully identified, that smoker referred the research team to a fellow marijuana smoker until the required number of participants for a focus group was met. This approach was adopted because marijuana smokers did not want to be publicly known by their non-smoking peers but were, however, comfortable to reveal their identity in the midst of other marijuana-smoking adolescents whom they trusted. Thus, the key informants, together with the first recruited adolescent marijuana smoker for each focus group, helped to identify subsequent participants with the required characteristics (marijuana smoking). Participants were therefore recruited and interviewed in secrecy, at secluded places, at the blind side of their legal guardians, community members and members of other focus groups.

### Data collection

An in-depth semi-structured FGD guide developed was used to collect the data for the study. The guide contained questions on four broad areas: socio-demographic characteristics of participants, personal, micro- and macro-level factors that influenced marijuana use initiation. The guide was piloted on five participants recruited from a community with similar characteristics as the two study communities.

The pilot study was used to evaluate and revise the interview questions to ensure trustworthiness of the instrument.^26^ The actual data collection commenced by following the necessary community entry processes because of the sensitive nature of the study. Data collection was preceded by community entry. During the community entry, permission to conduct the study was sought from the chiefs and ward councillors. After obtaining permission from community leaders, arrangements were made with them for community deliberations through community forums because of the sensitivity of the study. This was to ensure the safety of the research team during data collection process.

After obtaining permission from community members, data collection proceeded through formation of focus groups. Two focus groups per community, using already established key informants, were formed. The data were collected by the principal investigator (E.M.) who was a PhD researcher, under the guidance of two extensively experienced qualitative researchers, together with two trained research assistants in qualitative research within 1 month, in March 2016. In each focus group, unique identifiers (e.g. A1/C1) were assigned to the participants and were used to identify them in relation to their bio-demographic characteristics, communities, focus groups and contributions during the discussion. Each discussion lasted between an hour to one and half hours. The discussions were recorded with an Olympus voice recorder after seeking permission from the participants.

### Data analysis

Thematic content analytical techniques were followed to analyse the data. The voice recordings were first translated and transcribed from the IsiXhosa Language to English by a qualified language translator from the Eastern Cape Department of Basic Education, South Africa. Content analysis describes an analytic process that applies intuitive and interpretive approaches to systematically summarise textual data.^27^ The data sets were first labelled by E.M. to keep track of their source, thus the communities as well as the focus groups they came from. We (E.M. and M.D.) then thoroughly read through the various transcripts to gain a general sense of the information. Each transcript was put into segments and codes, which were descriptive in nature, in terms of the subject matter in the transcript. Coding was performed by writing the applicable codes in the margins of transcripts. Coding was performed by E.M. and M.D. indecently. The first author and second authors then reviewed discrepancies in the double-coded interviews and revised discrepancies through consensus by using the most expressive words for each set of codes. We then grouped related topics under various themes, based on already existing thematic areas reported in the literature,^2,28,29^ designated as deductive codes as well as those that emerged from the data analysis process and were unique to this study, referred to as inductive codes.^30^ Themes were identified by the first and second authors (E.M. and M.D.) and reviewed by the rest of the authors. Data saturation was deemed to have been achieved when there was non-immergence of new codes or themes from the transcripts. These themes were then used to structure the results of the study.

### Trustworthiness

#### Validity and reliability

The pilot study was used to evaluate and revise the interview questions to ensure validity and reliability of the instrument.^31^ The collected data were also given to peers with clear written instructions to check the transcripts to ensure validity and reliability and to categorise and develop themes from the data.^31^

#### Credibility, dependability, confirmability and transferability

Findings of our study were ensured to be credible, dependable, confirmable and transferable per the requirements of qualitative research.^24,32^ Credibility of our results was ensured by firstly staying in the communities for a month before data collection to gain the trust of participants to open them up for interviewing. Dependability was achieved by employing the services of peer and experienced qualitative researchers who constantly critiqued our processes and write-up for improvement purposes.^33^ We ensured that confirmability of the findings was achieved by reporting our initial transcripts and results back to the participants for verification, who then agreed that what we reported was indeed what they said. Lastly, we provided chronological detailed explanation of our methods and procedures which has ensured that our study could be replicated and thus our results becoming transferable.^34^

### Ethical consideration

Permission to conduct the study was granted by the Walter Sisulu University’s Faculty of Health Sciences, Human Research Ethics Committee (protocol number: 047/2013). Permission was also sought from all relevant stakeholders and thus community elders through community entry and community members through community forums. Parents also consented for their children below 18 years to partake in the study during the community forum discussions so long as participants agreed to partake in the study and their rights spelt out to them. Participation was voluntary and participant’s right to withdraw from the study was respected. Individual participants were assured of anonymity as alphabets were used to represent their identities, while numbers (1 and 2) were used to denote their respective communities. There were no direct benefits that discussants derived from the study nor were there risks associated with participating in the study.

## Results

We established 12 influences of adolescent marijuana use initiation, grouped under three main levels of socio-ecological influence: (1) personal characteristics (curiosity, shyness leading poor decision-making and fulfilment of personal need), (2) micro-level influences (peer pressure, negative school climate, presence of marijuana in households and parental or sibling marijuana use) and (3) macro-level influences (child labour, poverty, presence of marijuana in communities, presence of negative adult role models and breakdown in communal restrictions against marijuana use). The results, with their supporting quotes, were then presented under these themes.

### Socio-demographic characteristics of participants

All the 37 participants were men and were aged 14–19 years. Thirteen were in the age category of 14–16 years and 24 were in the age bracket of 17–19 years. There were two focus groups per community, making four in total. Community 1 had 19 participants, 9 in the first focus group and 10 in the second focus group. Community 2 had 18) participants with each group having nine members. Cumulatively, 31 participants had been to secondary school and the remaining six were primary school dropouts. All participants were living under the care of their parents or with a guardian. None of the participants was formally employed.

### Influences of adolescent marijuana use initiation

When participants were questioned about what influenced them to use marijuana for the first time, varied reasons were given and are grouped under three socio-ecological level of influence: personal characteristics, micro-level influence and macro-level influence.

#### Personal characteristics

Three personal-level influences, curiosity, shyness and fulfilment of personal need, were found to be associated with adolescent marijuana use initiation.

**Curiosity:** Some participants, out of their own curiosity, wanted to find out how it felt when one smoked marijuana. They had access to the marijuana plant and decided to experiment with it to know the feeling associated with marijuana smoking. This category of marijuana smokers was not directly introduced to smoking by anybody neither were they under any social pressure to smoke marijuana. Thus, they did so on their own volition. The following quotes summarise such participants’ views:

‘[*A*]s for me, I wanted to know how it felt when you smoke dagga [*marijuana*]. I have heard people talk about it [*feeling*] but have never experienced it so I needed to find out. One day when I was alone in the house I tried it and it felt good so I could not stop smoking afterwards’. (Participant 6, male, student, 16 years old)

‘[*I*] kept seeing [*young*] boys going to the bushes to smoke during break time [*at school*] and would come back to be bragging of how nice it is [*to smoke*] so as a [*circumcised*] man, I decided to experience what I was missing out on by smoking, so I can say that is how I started smoking [*marijuana*]’. (Participant 24, male, student, 17 years old)

**Shyness:** Some participants indicated that they were shy and did not have the courage to take certain decisions on their own and had to resort to marijuana use upon advice from their friends. They explained:

‘[*I*] was a shy guy and could not be bold to take decisions or do things for myself, so my friends always influenced the way I did things. I will say that I took a lot of bad decisions [*like marijuana smoking*] from my friends that did not help me. I was very good [*brilliant*] at school but because of this thing [*marijuana smoking*], I dropped out’. (Participant 9, male, student, 17 years old)

‘[*T*]he problem is that when I was young, was not able to talk to ladies, and I used to listen to my friend a lot. So I was told that if I should smoke dagga [*marijuana*], I will get the courage to approach girls [*laughs*] and I started smoking’. (Participant 31, male, secondary school graduate, 19 years old)

**Desire to belong to social group (sense of belonging):** Some participants indicated that they smoked marijuana to fulfil a personal need, thus to ‘belong’ in their group (sense of belonging). As they were in constant interaction with their peers who smoked, even though they were not urged to do so, they felt that it was necessary to smoke in order to ‘belong’ to the group. This urge of belonging pushed some participants to initiate marijuana smoking on their own, as captured by the quote below:

‘[*Y*]ou know, when you move with your friends and whenever they are about to smoke you just say ‘nah, I’m not part of this’, then it’s like you are not truly part of the group. So to prove to yourself that you are a true member of the gang, you have to do what they do, that is to smoke [*marijuana*]’. (Participant 15, male, student, 16 years old)

‘[*M*]y team mates were always smoking together and happy and I also wanted to belong’. (Participant 28, male, student, 18 years old)

#### Micro-level influences

Four micro-level influences were found to have initiated some adolescents into marijuana use, as per the data. These influences include pressure from peers, studying in negative school environments conducive for marijuana use, living in negative physical family environments where marijuana is available and having negative proximal family members who smoked marijuana as role models.

**Peer pressure:** Some participants explained that they were initiated into marijuana use by their peers who smoked marijuana. In order to be accepted by their social networks, they had a social responsibility to play by obliging to use marijuana. Thus, they smoked marijuana to fulfil a pressing social need which is conformity to group culture. The quote below summarises participants’ view:

‘[*I*] can say it is peer pressure because when you stay with friends who smoke, they also want you to try it [*smoke*] to experience the feeling they get. That is how I ended up smoking it [*marijuana*]’. (Participant 10, male, student, 17 years old)

‘[*W*]hat made me start smoking marijuana were my friends. As I was playing soccer [*as young adolescent, my team mates* (*peers*) [*sic*]] were smoking it [*marijuana*] and they will toss me a piece [*of marijuana*] so it was peer pressure [*that made me to smoke*]’. (Participant 26, male, secondary school graduate, 18 years old)

**Negative school climate:** The school environment also influenced some participants to smoke marijuana. Participants who had free time on their hand at school because of teacher absenteeism used such times to experiment with marijuana instead of learning. Moreover, in schools where children saw teachers smoking marijuana felt that it was a good habit to emulate and ended up smoking marijuana. Two participants explained:

‘[*E*]ish, brother, this is an interesting one [*question*]. You know, during my primary school days, If I can remember well… when I was in class four, our teacher was very lazy, he was always absent so we were always playing outside. Sometimes we just enter the bushes and the older ones among us will just light dagga [*marijuana*] for us to smoke’. (Participant 16, male, student, 14 years old)

‘[*I*] remember we also used to have this teacher called Mr…, he was smoking [*marijuana*] so much but he also used to like me a lot. So seeing him smoking marijuana at school made some of us to think that it is a cool thing to do some of us also started smoking’. (Participant 35, male, junior secondary school graduate, 17 years old)

**Presence of marijuana in households (negative physical family environments):** Some participants grew up in families where the physical environments exposed them to marijuana and therefore made it easy for such participants to start smoking at an early age. Some parents stored harvested marijuana in plain sight of children, making it easy for curious children to have access to marijuana and experiment with it. Participants’ narratives are captured below:

‘[*I*]n our hose marijuana was always all over the place as that was the only source of [*family*] income. So I don’t even remember how it happened but before I could realise, I was an experienced [*marijuana*] smoker’. (Participant 11, male, student, 18 years old)

‘[*I*]n my family, nobody teaches anybody how to smoke marijuana. The leaves are dried in the house and it is our [*children’s*] duty to watch over it. So as you keep doing this [*drying marijuana*], you end up smoking it without knowing and nobody will even complain’. (Participant 29, male, secondary school graduate, 18 years old)

**Parent and sibling marijuana use (negative family role models):** Being in the company of a close family member such as sibling, smoking marijuana was another key micro-level influence of adolescent marijuana use initiation as some participants took cues and directives from their elder siblings who smoked marijuana. Participants from both communities explained:

‘[*I*] would say that my brother influenced me to smoke [*cannabis*] because he was smoking all over the place, even in the house so I thought it was a cool [*good*] thing to do. I remember one day as he [*brother*] dropped a piece [*of marijuana*], I just took it and smoked it. That’s how I got involved in this thing [*marijuana smoking*]’. (Participant 9, male, student, 17 years old)

‘[*I*] used to smoke it [*marijuana*] when I went herding the cattle with my [*elder*] brother and when it was cold, he would smoke it [*marijuana*] would advise us [*the younger siblings*] to smoke as well so as to get warm. It later became hard [*for me*] to wake up and not smoke. My father also smoke, in fact, all the men in my family smoke it [*marijuana*]’. (Participant 33, male, secondary school graduate, 19 years old)

#### Macro-level influences

Five macro-level influences were found to have initiated discussants into marijuana use. They include: using children as a source of labour on marijuana fields, poor socio-economic background of children, neighbourhood characteristics, negative adult role models in communities and breakdown in communal law enforcement on adolescent marijuana use.

**Involvement of children in marijuana activities (child labour):** The use of children and young adolescents by parents and guardians as a source of cheap labour on marijuana plantations was found to be one of the macro-level factors that influenced participants into marijuana use. Children are often used to clear weeds on marijuana plantations, harvest, transport or dry marijuana for their families. This sometimes tempted some children to experiment marijuana smoking. Some participants’ explained:

‘[*I*] started working on marijuana plantations when I was [*very*] young. We [*the children*] were planting the seeds and also carrying it [*marijuana*] home after harvesting. Also when it is being dried [*at home*] we [*the children*] watched over it so the smoking came by itself’. (Participant 1, male, student, 18 years old)

‘[*I*]t is not easy to abstain from marijuana smoking, especially when you are involved in its activities so young at home. As a child, although you don’t have the energy like the adults, you also have a role to the play. For instance, I was carrying manure and also carrying harvested dagga [*marijuana*] back home so it is just a matter of time that you smoke it [*marijuana*]’. (Participant 6, male, student, 16 years old)

**Poor socio-economic conditions (poverty):** Poverty was another macro-level factor that pushed adolescents into marijuana use. Almost all participants came from poor socio-economic backgrounds, and thus, illegal marijuana cultivation and trading was the only source of family income for many. This exposed children to marijuana at early age and influenced their drug use habits. The excerpts below explain participants’ views:

‘[*S*]ometimes you cannot blame us [*marijuana smokers*] because there are no [*formal*] jobs here [*in the communities*] and we [*community members*] need to survive. The government doesn’t think about us [*the villagers*] so the only work we can do is to grow dagga [*marijuana*]. Our families have been doing this [*marijuana business*] for long so you also grow to join [*the family business*]. And how can you be working with dagga [*marijuana*] for such a long time as a boy without trying [*smoking*] it?’ (Participant 12, male, student, 15 years old)

‘[*I*] started smoking marijuana because of poverty. It [*poverty*] is so sever here to an extent that sometimes what to eat after school becomes a problem so they [*peers*] introduce you to dagga [*marijuana*] to deal with the hunger’. (Participant 37, male, student, 17 years old)

**Availability and accessibility of marijuana in communities:** A common cause of adolescent marijuana use initiation, as described by participants, was the availability and ease of accessibility to the plant in the communities. The plant was either cultivated or sold in both communities. As young adolescents saw marijuana grown and traded on regular basis, they already knew what it was used for and anticipated smoking at some point in the future. Below are responses of some participants:

‘[*D*]agga [*marijuana*] is available here and I knew what it was used for as a child so it was easy for me to smoke it, but I can’t remember exactly how I started to smoke’. (Participant 7, male, secondary school drop-out, 16 years)

‘[*T*]he problem is that if you want to smoke “nkantini” [*marijuana*] you easily get it because it is all over the place. Even if you don’t have the money to buy it, you can get it for free. I remember I used to steal it when my parents were not at home, by the time they knew that I smoke, I was already experienced in it’. (Participant 35, male, student, 17 years old)

**Presence of negative adult role models (marijuana smokers) in communities:** Imitation of adult marijuana smokers in the community, outside of participants’ homes, was another key macro-level construct that initiated adolescents into marijuana smoking. Participants saw individuals they revered in their communities openly smoking marijuana on the streets and followed suit, as captured in the following quotes:

‘[*T*]here is no one who taught me. Ok, …let’s say that as a child you see your neighbour or someone you know smoking it and when you ask them they say it is a medicine so you also want to smoke it to see how it feel’. (Participant 18, male, student, 18 years old)

‘[*T*]he problem is that people I knew and respected in the community were busy smoking it so I thought it was cool [*to smoke*]. So nobody directly taught me how to smoke dagga [*marijuana*], I just watched and learned from others’. (Participant 22, male, student, 14 years old)

**Breakdown in communal restrictions against marijuana use:** Another macro-level influence of adolescent marijuana use initiation was lack of communal enforcement of laws that abhor adolescent marijuana smoking. Adolescent marijuana smokers were not admonished or reprimanded by community members in any way when found smoking. This emboldened children, including adolescents to freely smoke marijuana, as narrated by some participants:

‘[*H*]ere (in this community), nobody cares whether you [*as a child*] smoke dagga [*marijuana*] or not. The thing [*problem*] is that everybody has it [*marijuana*] in his or her home, so if you see a child smoking, whom are you going to report it to? But maybe if the elders are bold to say that boys [*uncircumcised males*] should not smoke it that could work’. (Participant 2, male, secondary school graduate, 19 years old)

‘[*I*] will say that these days the community leaders have lost their power. It is not like before when they could punish children in public. Now there is no enforcement of laws against child marijuana smoking because their parents will come and insult you for punishing their children. When I started to smoke, we use to hide from the elders although they sensed we were smoking. But these days, children are bold enough to smoke in town’. (Participant 30, male, student, 19 years old)

## Discussion

In this study, we investigated the socio-ecological factors that influenced adolescent marijuana use initiation in two marijuana-growing communities of South Africa, based on the constructs of the socio-ecological model of drug initiation by the UNODC.^1^ Twelve themes, grouped under three broad levels of influences, personal characteristics, micro- and macro-level influences, were found to have initiated participants into marijuana use. With reference to personal characteristics, curiosity to find out how marijuana smoking felt, shyness, leading to poor childhood decision-making, and personal desire to belong to a social group were found to have influenced participants into marijuana use. Curiosity, described by Racz^35^ as a kind of internal force, was found to be one of the driving forces behind adolescents’ marijuana use initiation. Thus, adolescents had a burning desire to ascertain for themselves the suspicions they had about how marijuana smoking felt, either based on accounts of what their peers had told them or based on observed actions of their marijuana-smoking peers. Pleasure seeking was therefore the driving force behind adolescents’ curiosity as they wanted to explore the imagined good feeling associated with marijuana smoking and emulated the actions of their peers. Curiosity therefore intrinsically motivated participants to emulate the actions of their marijuana-smoking peers.^36^

Another personal characteristic that was found to have influenced adolescent marijuana use initiation was shyness, as it led to poor childhood decision-making. It was found that adolescents who were shy in their early childhood were most often indecisive and therefore depended much on their peers for ideas. This led to some being easily influenced by their peers to indulge in marijuana smoking. For instance, male adolescents who were shy to talk to girls, probably because of poor socialisation skills, depended on their friends on how to be able to approach girls. Such participants were therefore not able to object to the bad ideas proposed by their friends and ended up smoking marijuana upon advice from their friends. Explaining how shyness could influence substance use in similar contexts, Rothman et al.^37^ found that children who wanted to gain courage and be able to take up adult roles in the United States reported to have resorted to early alcohol use as means of fortification to be able to perform such roles. Also, in Ethiopia, students cited poor socialisation skill as their reason for indulging in substance use as they wanted to gain the courage to carry out their thoughts.^38^ Thus, when a person feels overwhelmed or incapable of performing an activity, they tend to look for a source of encouragement and may resort to substance use, either on their own volition or upon advice from their peers.^39^ Hence, parents and guardians should have frequent conversations with their children to know their weaknesses and help them address such weaknesses so that they do not resort to illicit substances as a way to address such a weakness.^40^ Beyond poor social skills, shyness could be clinical or sub-clinical manifestation of social anxiety. It has been empirically established that shyness leads to social phobia and avoidant personality disorder which some individuals try to address through self-medication by resorting to psychoactive drug use.^41^ As such, discussants who suffered from shyness, could in actual fact, might have been suffering social anxiety and thus resorted to marijuana use as a form of self-medication.^42,43^

Moreover, the urge to fulfil a personal need such as the need to belong to a social group strongly motivated some participants to use marijuana. This was the case for adolescents who had marijuana-smoking peers. Although they were not forcefully enticed by their peers to smoke, they felt that they did not truly belong in the group whenever their friends were smoking. Hence, to convince themselves that they were true members of the cult, they voluntarily decided to initiate themselves into marijuana smoking. Sense of belonging has been found to be cherished by members of a social group as it leads to group cohesion.^44^ In adolescent social networks, group cohesion is paramount as it brings about formation of intense relationships that create a safe and non-judgemental environment where members can feel cared for and accepted.^44^ Hence, there is some kind of innate pressure on group members to conform to group norms in order to belong and feel accepted.^45^ This craving for acceptance therefore influenced some adolescents to self-initiate marijuana use. With respect to micro-level influences of marijuana use initiation, direct peer pressure was found to be one of the motivating factors that influenced participants to use marijuana for the first time. As some discussants played sport with their marijuana-smoking peers, they were directly influenced to smoke marijuana. This happened when marijuana smokers intentionally gave their non-marijuana smoking peers a piece of marijuana to smoke.

The influence of peer pressure on substance use, including marijuana use, has extensively been reported in the literature. For instance, different studies have mentioned peer pressure as a strong driving force behind adolescent substance use, including marijuana.^46,47^ This is because as children grow, they form and join social networks. These networks have unique culture and etiquettes. Hence, for one to be truly considered as a group member, there is the need to conform to such group norms and etiquettes.^48^ Individuals wanting to join such groups are therefore initiated into such groups by having to indulge in the behaviour(s) that the group is associated with,^48^ such as marijuana smoking. Hence, as discussants joined soccer clubs and groupings where marijuana smoking was the norm, they were obliged to go through with the behaviour.

School environment was another micro-level influence of adolescent marijuana use initiation that was found to have initiated some discussants into marijuana use in the present study. For instance, in schools where discussants had much free time on their hands to play instead of being in the classroom, it created an opportunity to smoke marijuana by following marijuana-smoking peers into surrounding bushes. It has been reported that when school children are left unattended or unengaged, they are likely to use the freedom they have to indulge in health damaging behaviours such as marijuana use as an antidote to boredom.^49^ Also, school environments which are not well secured, or where teachers are constantly absent, promote marijuana use by giving students the opportunity to indulge such practices, especially in contexts where illicit drug activities are rife.^50^ Hence, school children should not remain unoccupied or unsupervised by a teacher or an adult at school in order not to have excessive free time that they could exploit to use substances.^51^ It was also found that schools where teachers serve as negative role models by smoking marijuana easily influence children to emulate such behaviour. This is because children often look up to their teachers as role models.^52^

Moreover, negative physical family environments, thus family environments in which marijuana was present, encouraged discussants to initiate marijuana use, as they felt permitted because of its presence. This was so because there were instances where marijuana was stored in the full glare of children in homes and were sometimes made to dry it, making it easily accessible to children to experiment with. The availability of marijuana in homes and environments put discussants in compromising situations by increasing their likelihood to use it.^42,53^ It has been found that children who grow up in households where marijuana is readily available often use marijuana as a drug of choice for substance use initiation, as opposed to children who grow up in contexts where marijuana is not easily available in their homes.^52^ Thus, while adolescents in rural settings such as our study participants often have easy access to marijuana, making it seems legal to them, those in urban settings see as illicit and hard to come by. Similar findings have been made in Morocco, where adolescents who grew up in households where marijuana is easily available used it.^29^

Furthermore, negative family role models, such as parent and or sibling marijuana use, were found to be another micro-level influence of adolescent marijuana use initiation. Some discussants lived in households where their parents and/or elder siblings smoked marijuana. Such discussants therefore saw marijuana smoking as an acceptable behaviour and learnt it. The problem of early marijuana use initiation in households where siblings or parent(s) use drugs has been scientifically reported. According to Kingston et al.^2^ early substance users reported initiation and use with older siblings and parents or guardians more often than later users. Hence, as parents and elder siblings smoked marijuana in the presence of their much younger siblings, they may have seen it as a good behaviour to copy and initiated marijuana smoking, either on their own or on the advice of their siblings. According to McLaughlin and colleagues,^54^ children living with families where substance use is rife face challenges such as neglect and abuse, and therefore, tend to adopt adaptive coping techniques resorting to drug use, as found in this study.

With regard to macro-level influences of adolescent marijuana use initiation, the use of children as a source of cheap labour on marijuana plantations to undertake activities such as planting, watering, harvesting and transportation of harvested marijuana to the house, most often made children vulnerable to marijuana use initiation. While children have the right to be protected from illicit drug production and usage, as documented in international conventions and treaties,^55^ this was not the case for discussants as they were extensively used on marijuana plantations as a source of cheap labour. Meanwhile, discussants did not view their involvement in marijuana cultivation as a form of maltreatment or abuse. This could be as a result of marijuana cultivation and trading becoming a norm in the two communities, and hence, participants saw their involvement in marijuana-related activities as part of their roles and responsibilities.^56^

Another macro-level influence of adolescent marijuana use initiation, per discussants’ narratives, was poor socio-economic conditions. Because of high level of poverty in the area,^57^ most families indulged in illegal marijuana cultivation and trading in order to earn some income. Discussants were therefore drawn into illegal marijuana activities by their families to help raise the much needed family income, while some grown it for their personal gain. This, however, influenced participants to initiate marijuana smoking. The use of illicit substances such as marijuana among adolescents from poor socio-economic settings is a common phenomenon in Africa. In Nigeria for instance, people from poor socio-economic backgrounds drink alcohol either as a result of idleness or because of the nature of work they are involved in.^58^ Thus, poor socio-economic conditions make people to indulge in illegal practices such as marijuana production to make income and while those who do not indulge in such activities remain idle.

Furthermore, available and accessibility of marijuana in the communities was found to be another macro-level factor that influenced participants to initiate marijuana use. In both communities, open display and cultivation of marijuana, although illegal, was a common practice. Hence, even when participants did not have marijuana in their homes, they could get it for free in their neighbourhood. The neighbourhood environment therefore influenced participants to initiate marijuana use. The nature of neighbourhoods in which children grow plays a crucial role in the type of behaviour they adopt. Thus, in contexts where marijuana is easily accessible, adolescents are bound to use it, as was the case in Ethiopia.^38^ Neighbourhood characteristics have social influence on the development of a child.^59^ Hence, children who grow up in unsafe neighbourhoods, such as where drugs are easily available or where people are seen to be openly indulged in drug abuse, end up copying such behaviour.^47^ This is so because such behaviour is seen to be socially acceptable. As such, there is minimal effort by parents and other community members to curtail it.^2^

Moreover, the presence of adult marijuana smokers in the communities was another macro-level influence of adolescent marijuana use initiation. As young adolescents often look up to people they revere in their surroundings to emulate, marijuana smokers whom they revere in their communities served that purpose. Hence, the indulgence in open marijuana smoking by adults in the communities made participants to believe that marijuana smoking is a behaviour worth emulating. Similar findings have been reported from similar contexts. In Morocco, for instance, in situations where adolescents were eager to be like others in their communities, thus, people they revered as role models, they emulated their actions, including marijuana smoking.^29^ Thus, adult marijuana smoking had become a social norm in the communities and enticed participants to smoke. In contexts where parents and adult community members served as positive role models, it deterred children from indulging in bad behaviours such as marijuana use.^54^ It could therefore be said that the presence of negative role models and the lack of positive ones in our study communities influenced participants to initiate marijuana use.

Lastly, breakdown in communal restrictions against marijuana use, especially adolescent marijuana use, encouraged participants to initiate marijuana use. Living in communities where there is breakdown in social order, participants capitalised on the opportunity to smoke marijuana. Per their own narratives, there used to be communal ban on open marijuana smoking, including adolescence smoking. In those days, the elderly were at liberty to punish wrongdoing such as adolescent marijuana use. However, with passage of time, such social corrective mechanisms have broken down, allowing adolescents to freely indulge in marijuana smoking.

Minimal efforts by parents and other community members to curtail adolescent substance use have been identified as an influence of adolescent marijuana use initiation, whereby some parents and guardians approved of their children’s marijuana use.^2^ We also found that in situations where adolescents used marijuana in the presence of other community members, they were not admonished for such behaviour and allowed it to fester as it had become a social norm.^29^ Thus, communal laws or measures in place that frowned on adolescent marijuana use initiation.

## Limitations

Although measures were taken to ensure credibility and transferability of our findings, a major limitation of this study is that the findings cannot be extended to the wider South African adolescent population with a degree of certainty because the findings could not be tested to see whether they were statistically significant or because of chance, as in the case of quantitative research.^60^ Another limitation of the study is its sole focus on men as it failed to establish the perspectives of women on the subject. This was, however, because of difficulty in identifying and recruiting female marijuana smokers in the communities.

## Conclusion

Three key socio-ecological influences, personal, micro and macro-level influences, were found to be behind adolescent marijuana use initiation. We therefore recommend that health education and promotion programmes targeting socio-ecological motives for adolescent marijuana use initiation in the two communities should be intensified to break the cycle of adolescent marijuana use. This could be achieved by implementing alternative livelihood schemes in the affected communities to break the cycle of illegal marijuana cultivation that ecologically exposes and influences adolescent marijuana use initiation.
